# Copper-Alloy Surfaces and Cleaning Regimens against the Spread of SARS-CoV-2 in Dentistry and Orthopedics. From Fomites to Anti-Infective Nanocoatings

**DOI:** 10.3390/ma13153244

**Published:** 2020-07-22

**Authors:** Claudio Poggio, Marco Colombo, Carla Renata Arciola, Tiziana Greggi, Andrea Scribante, Alberto Dagna

**Affiliations:** 1 Department of Clinical, Surgical, Diagnostic and Pediatric Sciences—Section of Dentistry, University of Pavia, Piazzale Golgi 2, 27100 Pavia, Italy; marco.colombo@unipv.it (M.C.); alberto.dagna@unipv.it (A.D.); 2 IRCCS Istituto Ortopedico Rizzoli, Laboratorio di Patologia delle Infezioni Associate all’Impianto, via di Barbiano 1/10, 40136 Bologna, Italy; 3 Department of Experimental, Diagnostic and Specially Medicine, University of Bologna, via San Giacomo 14, 40126 Bologna, Italy; 4 IRCCS Istituto Ortopedico Rizzoli, Chirurgia delle Deformità del Rachide, via di Barbiano 1/10, 40136 Bologna, Italy; tiziana.greggi@ior.it

**Keywords:** SARS-CoV-2, COVID-19, copper, Cu nanoparticles, environmental contamination, healthcare-associated infection, hygiene, dentistry, orthopedics

## Abstract

The latest diffusion of severe acute respiratory syndrome coronavirus 2 (SARS-CoV-2), responsible for the coronavirus disease (COVID-19), has involved the whole world population. Even if huge efforts to control the pandemic have been done, the viral spread is still continuing. COVID-19 is reported as a zoonosis jumped from bats and pangolins to humans. After infection in humans, SARS-CoV-2 is found in the nasopharyngeal and salivary secretions. The virus has also been detected in the blood plasma of infected patients. The viral spread occurs through droplets exhaled from the nose and mouth of the infected people when they breath or talk, or through droplets propelled as a dense cloud by chough or sneeze. The virus can also be delivered as an aerosol from blood plasma, through surgical procedures. Following these ways, the virus can disperse in the air, then reaching and settling on the exposed surfaces. How long the virus will survive on a surface depends on the material the surface is made from. Infection via high-touch surfaces should be prevented. Copper alloy coatings, combined with efficient hygienic/disinfectant procedures and careful surgical practice, could be helpful to health protection in dental practice and can also be adopted in orthopedic traumatology.

## 1. Introduction

Viruses can live on many surfaces outside the human body and persist on inanimate surfaces like metal, glass or plastic for days [1]. SARS-CoV-2 (acronym for severe acute respiratory syndrome coronavirus 2), responsible for the current outbreak that causes COVID-19 (acronym for “corona virus disease 2019”), is reported to be able of surviving on inanimate surfaces for days. Although person-to-person transmission (through the droplets emitted by infected people when they breath, talk, cough or sneeze) has been referred as the primary way to spread the virus, however an indirect way of infection from contaminated surfaces has also been postulated. This occurs when an infected individual contaminates a surface. An uninfected individual who touches the contaminated surface will transfer viruses to his/her hands. Then, touching nose, eyes or mouth, he/she will carry viruses to his/her mucous membranes, thus causing self-inoculation.

It can therefore be said that SARS-CoV-2 spreads through respiratory droplets, but also through contaminated surfaces [2].

These disease transmission routes were astoundingly recognized centuries ago, back when the knowledge of microbes didn’t exist. In 1546, Girolamo Fracastoro in his “De contagione et contagiosis morbi” conceived for the first time that healthy people can get sick for direct contact with sick people or by touching objects belonging to sick people. He gave these objects the definition of *fomites*, that is, fuses that can transmit disease [3]. In 1897, Carl Flügge demonstrated for the first time that the droplets exhaled by a sick individual were capable of transmitting disease. For this reason, the respiratory droplets are known as “Flügge’s droplets” [4].

Since its emergence in December 2019, COVID-19 has impacted several countries all over the world, affecting thousands of patients and becoming a global public threat. It is therefore of utmost importance to prevent any further spread in the public and healthcare centers, in order to control the pandemic [5].

In dentistry, most procedures generate significant amounts of droplets and aerosols: dentists and patients can be exposed to pathogenic microorganisms, including viruses and bacteria that infect the oral cavity and respiratory tract. In dental practice, the transmission of microbial pathogens and, among these, of viruses can occur through different ways. By direct contact with patient’s secretions or tissues is a first way: indeed, patient’s secretions (such as saliva), fluids (transudates, exudates), blood, or other biological materials carry a viral bioburden. A second way is by inhaling airborne virus. In fact, SARS-CoV-2 was found to remain viable in aerosols up to three hours, although with a 1 log reduction in infectious titer [6]. There are two other possible ways of transmission: contact of mucous membranes with droplets and aerosols propelled at distance by an infected individual, when he/she breaths, talks, sneezes or coughs without wearing a mask; and indirect contact with contaminated instruments and surfaces that act as passive vectors, the so called *fomites*.

Impressively, the most turbulent exhalation clouds, with their great propulsive force, can reach up to 8 m away [7].

The risk of SARS-CoV-2 spreading in daily dental practice is high, because of the direct contact of the operators with infected asymptomatic patients. By in vitro cultures, live viruses were detected in the saliva of the infected individuals [8,9]. Moreover, the practice of dentistry involves the use of rotary instruments, handpieces, ultrasound-based instruments and air-water syringes: the use of these devices produces a cloud of droplets, saliva, blood, and debris, along with microorganisms.

**Figure 1:**
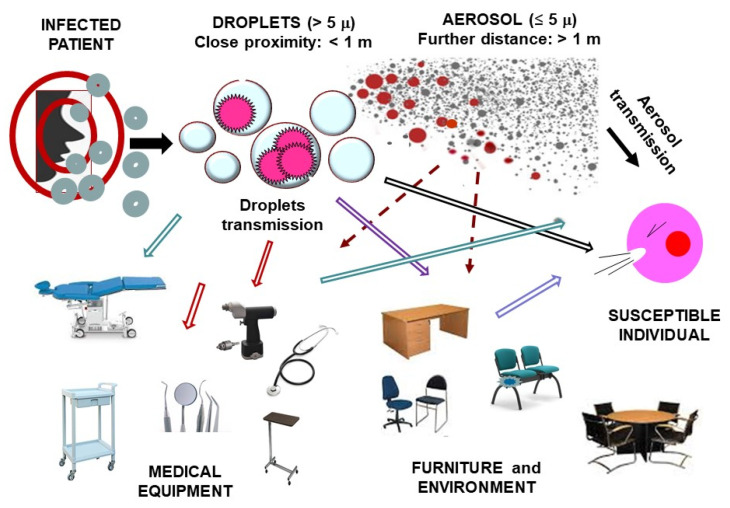
Routes of transmission in outpatient practice (adapted from Ge et al. [15]).

This cloud takes a short journey, then it settles on the nearby operating surfaces, and it falls on the floor or back on the patient himself, or it covers the dental health personnel. As showed in Figure 1 the spread via contaminated surfaces should also be considered.

In the dental environment, the microorganisms’ cross-transmission between inanimate surfaces and patients or healthcare workers can lead to healthcare-associated infections [10].

The knowledge of mechanisms of the aerosol generation and virus spread can favor the recognition and the amendment of the mistakes done in daily dental practice. In addition to the standard hygienic/cleaning protocols, further stringent and appropriate precautionary measures are required during the actual outbreak [11].

Guidelines and recommendations for limiting the transmission of SARS-CoV-2 in dentistry specialty have been proposed. These guidelines are reviewed in [12,13].

Besides dentistry, also orthopedics—mainly orthopedic traumatology—is a surgical specialty that entails procedures at high risk to generate aerosols.

In this connection, SARS-CoV-2 can be spread from body tissues and fluids of an infected patient to the environment, thus reaching care workers and surfaces [6]. This can occur following orthopedic traumatology procedures that involve incising tissues, truncating vessels, cleaning and irrigating wounds, electro cauterizing bleeding tissues, treating broken bones and injured areas around broken bones, realigning limb, reaming bone, drilling bone, aspirating hemorrhagic synovial fluid after traumatic hemorrhage [14].

Genetic material of SARS-CoV-2 has recently been demonstrated in the plasma of patients with COVID-19, thus feeding concerns for virus shedding during surgical procedures [16].

In a recent study, the prevalence of orthopedic surgeons who had become infected with SARS-CoV-2 in Wuhan (China) hospitals was found high. Exposure to viral infection was traced. Orthopedic surgeons operating in the wards were exposed to the highest risk of infection (close to 80%). Interestingly, the public places of the hospital turned out to be areas at high risk of infection (over 20%), even higher than that associated with operating rooms (12.5%) [17]. It should be emphasized that during the viral epidemic, orthopedic trauma services remain necessarily open and active, contributing to the chances of the virus to spread from asymptomatic infected patients who come to the observation of orthopedic surgeons [18]. The risk of Covid-19 infection in orthopedic practice and the personal protective equipment have been recently considered [19,20].

## 2. Persistence of SARS-CoV-2 on Inanimate Surfaces

Recent studies have shown that SARS-CoV-2 can survive from hours to days on a multiplicity of artificial surfaces like metal, glass or plastic (see Table 1). Kampf et al. [21] states that human coronaviruses can persist for up to 9 days at room temperature, and that veterinary coronaviruses can even persist for up to 28 days. It is noteworthy that increasing temperatures to 30 °C or more can shorten coronavirus survival, even if there is no established correlation between the variations in temperature and the spread of coronavirus [22]. The life span of the virus on a surface depends on many factors, including the temperature, humidity and ventilation of the room and the type of surface [23]. This denotes that people may contract the virus and become infected by touching an infected object [24].

The load of SARS-CoV-2 on non-living surfaces (fomites) is still unclear and probably variable, but the disinfection/cleaning of the surrounding surfaces appears to reduce the viral load, especially that of the objects touched by the infected person and the nearby area, where the maximum viral load is present [25].

A person can be infected with the coronavirus by touching a surface or an object that has viral particles on it and then by touching mouth, nose, or eyes [24]. Face-touching is a common habit of people: it is considered a vector in self-inoculation. An interesting 2008 article that dealt with environmental hygiene focused on the importance of the transmission of respiratory tract infections through the contact of contaminated hands with face. The article pointed out that a not small portion of human respiratory tract infections is transmitted through this path. Infection is likely to occur at a rate proportional to the number of contacts the hands have with the facial areas, in particular with the facial mucous membranes [25]. Frequent hand hygiene, both before and after patient contact, is an effective way to reduce colonization and limit transmission of infection for themselves and for patients: washing hands and not touching face are the best ways to minimize the chance of picking up the coronavirus from surfaces [24].

Van Doremalen et al. [26] investigated the survival of SARS-CoV-2 in the air and on surfaces: they simulated how virus could be spread by infected people onto common surfaces in domestic or hospital sceneries, by coughing, sneezing or touching/handling objects. They also explored how long the virus remained infectious on these surfaces. They tested viral vitality on plastic, stainless steel, copper, and cardboard. They also simulated an aerosol suspension with the virus, creating a mist of tiny droplets: in that way, it was possible to determine if the virus could remain in the air and how long. Under these experimental conditions, SARS-CoV-2 showed to be active and infectious on plastic and stainless-steel surfaces from 2 to 3 days, on cardboard for up to 24 h, and on copper for 4 h.

SARS-CoV-2 virus was detectable in aerosols for up to 3 h [23]. These times should vary in real conditions, due to the influence of factors such as temperature, humidity, ventilation, dust, fingerprints, organic debris, and the amount of virus deposited. Additionally, the characteristics of the surface must be taken into careful consideration: generally, smooth surfaces are more easily sanitized. On the other hand, the difficulty in cleaning increases with the roughness of the surface, as debris could penetrate the cracks and resist traditional alcohol-based cleaning treatments. These results obviously imply that people can catch the SARS-CoV-2 through the air and after touching contaminated objects. For these reasons, frequent disinfection of touched objects and surfaces play a fundamental role in reducing viral cross contamination [24].

In a recent article all the sources currently present in the literature concerning the persistence of the different coronaviruses in the environment as well as in medical and dental settings have been described and critically analyzed [27].

## 3. Inactivation with Biocidal Agents

World Health Organization (WHO) advises frequent, correct and consistent disinfection/cleaning procedures of environments and surfaces, which must be thoroughly cleaned with water and simple disinfectants [29]. Hospital disinfectants such as alcohol or bleach-based products are considered effective. These products must be used following scrupulously the manufacturer’s instructions. Particular attention must be paid to touched surfaces such as doors, handles, toilets, desks, switches and sinks: these surfaces can be frequently disinfected and cleaned with household disinfectants. There is a not negligible probability of environmental contamination. Since the potential for viral transmission through inanimate objects is strong, every effort should be made to consistently and effectively clean and disinfect surfaces and objects. However, these findings do not change the cleaning practices for coronaviruses, including recommendations issued by the US Centers for Disease Control and Prevention (CDC) [30]. It is essential that appropriate protocols for daily cleaning and disinfection are part of the measures to prevent infection. Cleaning procedures remove large numbers of microorganisms from surfaces and should always precede disinfection (a less lethal anti-microbial process than sterilization). Routine cleaning and disinfection can achieve the removal and elimination of the main pathogenic microorganisms, although not necessarily of all microbial forms (e.g., bacterial spores) [27,28].

The role of cleaning and disinfection is more critical for surfaces of medical offices that are more exposed to pathogens, including clinical contact surfaces (e.g., frequently touched surfaces such as light handles, bracket trays, switches on electrical equipment, dental units, computer equipment) in the patient-care area. When these surfaces are touched, even if the operators pay their attention, microorganisms can be transferred to other surfaces, instruments or to the nose, mouth, or eyes of healthcare personnel or patients. Although hand hygiene is the key to minimizing the spread of microorganisms, clinical contact surfaces should be protected by single-use barriers or cleaned and disinfected between patients. For the disinfection procedures, the American Environmental Protection Agency (EPA)-registered hospital disinfectants or the detergents/disinfectants labeled for use in health care settings should be used. It should also be noted that a disinfectant product can be used as a detergent only if the label explicitly indicates that the product is suitable for that use. The environmental infection control measures for SARS-CoV-2 are not unexpected. The management of SARS-CoV-2 in health care settings (especially in those patient-care areas in which aerosol-generating procedures are conducted) involves a strict adhesion to the manufacturer’s recommendations for routine cleaning and disinfection, for example the use of water and cleaners to pre-clean surfaces before applying disinfectants. Additionally, the use of dedicated medical equipment, as FFP2 or N95 face masks, are needed [28].

Different types of biocidal agents, such as alcohols, hydrogen peroxide, benzalkonium or sodium hypochlorite chloride, are applied worldwide for disinfection. It has been proven that disinfectants with 62–71% ethanol or 0.1% sodium hypochlorite can reduce coronavirus contamination on surfaces within one minute of exposure. Cleaning should be done after disinfection for contaminated surfaces.

Kampf et al. [21] reported that human coronaviruses could be inactivated on surfaces with ethanol (between 78–95%), 2-propanol (between 70–100%), the combination of 45% 2-propanol with 30% 1-propanol, glutardialdehyde (between 0.5–2.5%), formaldehyde (between 0.7–1%), and povidone iodine (between 0.23–7.5%). Sodium hypochlorite requires a minimal concentration of at least 0.21% to be effective. Hydrogen peroxide is effective with a minimal concentration of 0.5% and an application time of 1 min. Kampf et al. [21] have discussed the evidence on the disinfectant efficacy of benzalkonium chloride, which is still being tested and is not always effective. At contact times within 10 min, a concentration of 0.2% revealed no efficacy against enveloped human coronavirus, whereas a concentration of 0.05% proved quite effective against a non-enveloped coxsackie virus. Finally, using ozone is another potential effective measure to counteract viruses. Ozone can be conveyed as a gas or a gel to sanitize the environment. It has been extensively studied and used for many years and its effectiveness has been demonstrated against viruses, bacteria and fungi [28,31,32,33,34,35], even if no tests have been conducted on SARS-CoV-2 yet.

The important thing when disinfecting a surface is to bring the potential infectious dose of the virus below a level that would cause disease.

A correlation between SARS-CoV-2 burden and the severity of clinical course of the COVID-19 infection in five patients has been recently presented [36]. The authors described three different clinical types of evolution with detailed viral sampling: (1) two paucisymptomatic women, (2) a two-step disease progression in two young men and (3) an 80-year-old man with a rapid evolution towards multiple organ failure. The viral burden both in plasma and in the respiratory tract during infection was found correlating with the severity of infection. [34]. This interpretation has been discussed by Joynt and Wu, who have highlighted that the impossibility to differentiate between infective and noninfective (dead or antibody-neutralized) viruses remains a major limitation in establishing a correlation between the viral RNA burden and the severity of the infection [37].

## 4. Copper Alloy Touch Surfaces: An Effective Solution to Prevent Virus Spreading

The potential use of biocidal surfaces to provide constant antiviral activity against continual surface recontamination could help to limit the spread of respiratory viruses. Each material able to inactivate SARS-CoV-2 should be investigated and applied on critical surfaces in medical and dental offices, with the aim to reduce the number of viruses potentially deposed on devices, tools and furniture.

Copper has shown antiviral properties: the efficacy of the copper against bacteria and fungi is known since antiquity. For centuries, long before the knowledge about germs or viruses, people were aware of the disinfecting power of copper. In ancient times, Egyptian and Babylonian soldiers put the metal shavings of their bronze swords (made of copper and tin) into open wounds to reduce infection. Greeks, Romans and Aztecs used copper to treat headaches and ear infections. And in India, copper vessels have been used for millennia in the transportation of water [38].

The metal nanoparticles of copper, magnesium, titanium, gold, and zinc have been proved to be bactericidal at nano-levels [39]. New antiviral effective metal materials may be provided by the advancement of nanotechnology. The activity of the metal nanoparticles against viruses as an advanced -although still unexplored- field has been discussed in a noteworthy review [40].

Bacterial adhesion to the surface is the first step in the pathogenesis of implant infection, as the implant provides a surface to which bacteria can adhere. Modifying the implant surface with a repellent coating to reduce bacterial adherence can decrease the risk of infection. As observed since long ago heparin surface treatment of a material is effective in hindering bacterial adhesion [41]. In case of nanoparticles also it has been proposed that the differential antiviral effectiveness of silver or gold nanoparticles against different viruses depends on their surface functionalization with different ligands [42].

The antibacterial properties of copper chelates and copper nanoparticles have been tested against *E. coli, S. aureus* and *E. faecalis* [43,44]. Cu-amino acids chelate proved ten times more effective than Cu nanoparticles and Cu-EDTA chelate.

Currently, copper has been recognized as the best antimicrobial metal by the American Environmental Protection Agency (EPA). EPA has even approved the registrations of copper alloys as “antimicrobial materials with public health benefits” allowing manufacturers to make legal claims to the public health benefits of products made of registered alloys. The Agency has also approved a long list of antimicrobial copper products made from alloys, like bedrails, handrails, over-bed tables, sinks, faucets, doorknobs, toilet hardware, computer keyboards, health club equipment, and shopping cart handles [45].

In addition to the copper alloys, other hard surfaces embedded with copper oxide microparticles have been approved by the EPA [46] with demonstrated potent antimicrobial efficacy [44]. They are indeed in use in several hospitals in the USA [47]. These polymeric-based copper-oxide-impregnated surfaces are easier to incorporate and more adapt than copper alloys and thus their use in the context of dental clinics is very relevant.

Many functional nanoparticles with remarkable antiviral ability, such as quantum dots, gold and silver nanoparticles, nanoclusters, carbon dots, graphene oxide, silicon materials, polymers and dendrimers have been studied and the possible antiviral mechanism of action described. However, biocompatibility and biosafety issues in the use of nanoparticles are far from being solved [48].

**Figure 2:**
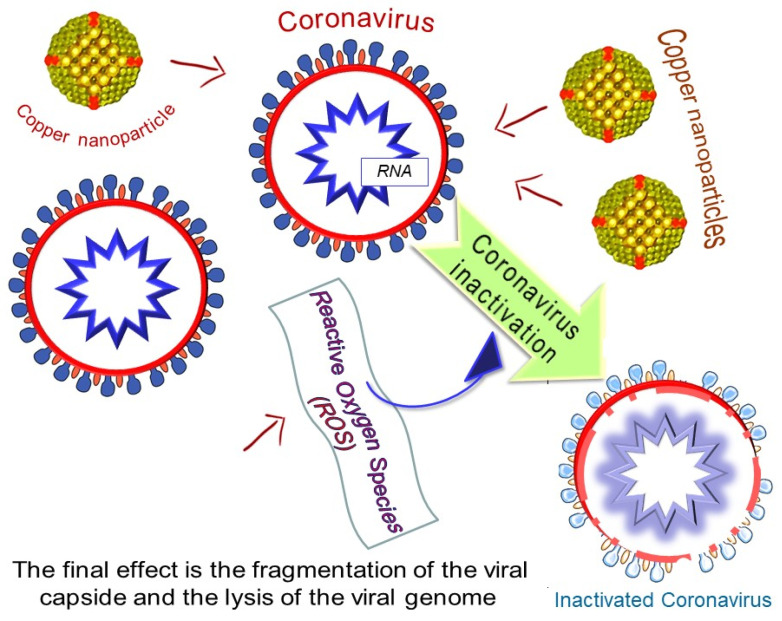
Coronavirus inactivation by copper nanoparticles.

Warnes et al. [49,50] investigated the efficacy of a range of copper alloys to inactivate Human Coronavirus 229E (HuCoV-229E). A rapid inactivation of human coronavirus occurs on brass and copper nickel surfaces at room temperature (21 °C): copper ion release and generation of reactive oxygen species (ROS) are involved in the inactivation of HuCoV-229E on copper and copper alloy surfaces. The final effect is the fragmentation of the viral genome, ensuring that inactivation is irreversible. Figure 2 gives a representation of the virus inactivation drawn from the knowledge of the effects of copper nanoparticles [48] and copper alloys [50] on the integrity of the viral capsid and viral RNA. Exposure to copper surfaces results in morphological changes to human coronavirus particles visible in transmission electron microscopy (TEM) [50].

Colin et al. [51] investigated the efficacy of copper alloy touch surfaces in healthcare facilities to prevent bacterial spreading and environmental bacterial contamination in healthcare facilities from inanimate surfaces to patients or healthcare workers. Dentists and other health workers, although working with gloves, can have a high risk of contamination following a direct contact with mouth of infected patient. Hygiene is for sure the first strategy to prevent healthcare-associated infections, but the professionals can touch many devices and surfaces in the operative room and viruses too like SARS-CoV-2 can spread through touch of surfaces. Indeed, by multiple surface-skin transfer, a contamination on a primary surface can somehow very quickly spread across an entire dental unit, or dental furniture, or medical clinic, or surgical service. A specific strategy to protect these surfaces may represent a way to limit the contamination for users and for patients, also considering that most of the microorganisms can survive on these surfaces for an extremely long time.

Copper shields or sheaths or coatings applied on critical frequently touched surfaces could be a solution. In fact, rapid inactivation, irreversible destruction of viral RNA, and massive structural damage were observed in coronavirus exposed to copper and copper alloy surfaces. Incorporation of copper alloy surfaces in conjunction with effective cleaning regimens and good clinical practice could help to control transmission of respiratory coronaviruses, including MERS and SARS [52,53].

Copper alloy is well-known for its antimicrobial properties and several studies have highlighted that copper alloy can break down the microbial load on the surfaces it coats and counteract infection in intensive care units. Recently, an effective antiviral activity by copper alloy against RNA viruses has been demonstrated. Copper acts against the virus with a dual harmful mechanism, on one side by degrading the viral RNA and on the other destructing the viral capsid, which would hinder the entry of copper [50].

The suggestion that emerges from these studies, which deserves to be emphasized, is that copper alloy surfaces could express antiviral activity against other RNA viruses that are believed to be able to spread from touch surfaces, including coronavirus (although not there are still published data for them) and the Ebola virus [50].

Colin et al. [51] also suggest the use of copper for door handles. And indeed, even the usual gesture of closing or opening a door by touching its handle causes microbial contamination, as well as a sharing of microbes with those who will touch the handle after us, and thus ultimately an indirect transfer of microbes from one individual to another. Copper handles can reduce the average bacterial burden up to 59% in respect to the traditional stainless-steel handles commonly utilized in hospitals, dental settings, schools, and other public places.

Inactivation of bacteria and viruses by copper relies on Cu^+^ and Cu^++^ hydroxyl radicals generated by an electrochemical process during aqueous corrosion of metallic copper [53,54]. In the case of copper alloys, copper ions in solution are produced from copper in the sequestered oxide layer formed over the surface of the alloy or released but chelated with some molecular species in solution [54]. Details of metallic corrosion processes producing biocide copper and the antimicrobial and antivirus effects of copper alloys are discussed by John R. Scully [55].

Another interesting aspect of copper application is through “nanoparticles” (NPs): they have been defined by the *Encyclopedia of Pharmaceutical Technology* as solid colloidal particles ranging in size from one to 1000 nm (one micron) [56]. NPs are endowed with multifaceted chemical properties and biological activities and promise many possible uses in the biomedical field. The evolution of metallic NPs has conducted to the development of a new family of antimicrobial materials. Highly ionic metallic NPs are of interest due to their extremely high surface areas and numerous reactive surface sites with unusual crystal morphologies. Cu-based NPs can be produced using different techniques (chemical treatment, thermal treatment, electrochemical synthesis, photochemical methods, sonochemical techniques). A new plastic antimicrobial agent including polypropylene with embedded Cu metal or copper oxide NPs was examined by Delgado et al. [53]. So, hopefully soon, Cu-NPs based materials could probably be used as effective antimicrobial systems in pharmaceutical, biomedical, and medical fields to combat pathogenic microorganisms.

In dentistry, the use of copper has been proposed as implant surface treatment [57], as endodontic irrigating solution [58], and as overlay for handles in dental offices [44].

Moreover, NPs lend themselves to being used in sterilizing medical devices, as well as in preparing chemical disinfectants or coatings.

However, more in-depth studies will be necessary to succeed in minimizing the toxicity of the metal and metal oxide nanoparticles and to apply them as safe antimicrobials in the medical field. Although they are effective against virulent microbes and have resistance to heat, their toxicity at higher concentrations is a limit. Toxicity-minimized NPs might be promising for controlling and treating different infectious diseases in the future [59,60].

## 5. Conclusions

The high care in cleaning and disinfecting all the devices, the furniture and the environment in medical, surgical and dental field, for daily practice, represents the key for guaranteeing the protection of professionals and patients. The new human coronavirus SARS-CoV-2 emerged in Wuhan, China, in late 2019 is now causing a pandemic. So, its diffusion may affect medical practice, due to its aerosol production and surface stability. Operators must protect themselves and must prevent cross-infections, minimizing the risk of new infections and paying attention to decontamination of dental offices and surgical services [61,62].

Dental professionals are exposed to high risk of contagion through patient’s saliva, blood, and aerosol/droplet during dental practice [61,62,63,64]. The use of handpieces under irrigation generate aerosol particles of saliva, blood, and secretions and facilitates the contamination of the environment and instruments, dental apparatuses, and surfaces [62,63,64,65,66,67]. SARS-CoV-2 can occur through inhalation of aerosol/droplets from infected individuals or by direct contact with mucous membranes, oral fluids, and contaminated instruments and surfaces [21,63]. Orthopedic surgeons too that operate in traumatology services are to be included among the professionals at high risk of generating aerosols. And indeed, orthopedists use surgical procedures that cause the spread of fluids, tissues and debris of biological materials, similarly to what happens for dental professionals in their practice and recommendations to protect the orthopedic and trauma surgeons and the personnel of the musculoskeletal health facilities have been recently published [68,69].

The available literature and actual clinical experience are still not able to suggest which protections to use when treating patients during COVID-19 pandemic in dental practice and in medical clinic, so each kind of device or shield or surface available has to be considered and evaluated [64,65,66,67].

Given this context, the use of copper surfaces brings a new perspective for constant and inherent disinfection. These copper surfaces already play an effective role in thwarting the viral spread, even in non-optimal use. According to the reports, metal and metal oxide nanoparticles represent a group of materials which were investigated in respect to their antimicrobial effects. In the present review, we focused on the recent research works concerning antimicrobial activity of metal and metal oxide nanoparticles together with their mechanism of action. Reviewed literature indicated that the particle size was the essential parameter which determined the antimicrobial effectiveness of the metal nanoparticles.

It was therefore proposed that in addition to the existing infection control armamentarium, the incorporation of copper into surfaces could effectively reduce environmental contamination, thus rendering healthcare facility surfaces safer in dentistry as well as in other surgical settings: anyhow, the use of antimicrobial copper is not a substitute for good hygienic practices. Other experimental evaluations on different surfaces or even on biological surfaces are certainly needed, to better understand the times of persistence of this virus and promote adequate standards.

## Figures and Tables

**Table 1:** Persistence of SARS-CoV-2 on different surfaces.

Material	Virus Strain	Coronavirus Persistence
WOOD	SARS-CoV, Strain P9	4–5 days [6,24,27]
GLASS	SARS-CoV, Strain P9	≤ 4 days [6,24,27]
PLASTIC	SARS-CoV, Strain HKU39849 MERS-CoV, Isolate HCoV-EMC/2012	≥ 5 days [6,24,27] >> 48 h [24,26]
PAPER	SARS-CoV, Strain P9	4–5 day [6,24,27]
CLOTHES	SARS-CoV, Strain GVU6109	2–4 days [26]
LATEX (surgical gloves)	HCoV, Strains 229E and OC43	≤ 8 h [28]
STEEL	SARS-CoV, Strain P9	3–5 days [6,22,28]
COPPER	SARS-CoV, Strain P9	≤ 4 h [6,28]
